# Exploring the Spectrum of Electrolyte Imbalances in Preeclampsia: Mechanisms, Implications, and Clinical Insights

**DOI:** 10.7759/cureus.67666

**Published:** 2024-08-24

**Authors:** Aishwarya Gupta, Dharmesh J Patel, Sandhya Pajai

**Affiliations:** 1 Department of Obstetrics and Gynaecology, Jawaharlal Nehru Medical College, Datta Meghe Institute of Higher Education and Research, Wardha, IND

**Keywords:** preeclampsia, pregnancy-induced hypertension, derangement, gestational hypertension, electrolyte

## Abstract

Preeclampsia, a complex and perplexing disorder unique to pregnancy, is widely recognized as primarily originating from placental dysfunction and can only be resolved by the delivery of the fetus in severe cases. Preeclampsia is a prevalent medical issue during pregnancy and is associated with elevated rates of maternal and infant mortality and morbidity. The exact cause of preeclampsia remains uncertain, although multiple factors have been implicated in its development based on current knowledge. Preeclampsia is characterized by maternal endothelial dysfunction due to the presence of fetal-derived circulatory substances from the placenta. The condition is associated with various risk factors, including maternal comorbidities such as chronic renal disease, hypertension (HTN), and obesity. Additionally, a family history of preeclampsia, nulliparity, multiple gestations, previous instances of preeclampsia, or intrauterine fetal growth restriction (IUGR) are considered risk factors. Electrolytes, including sodium, potassium, and chloride, play a critical role in the function of vascular smooth muscles and may potentially contribute to the pathophysiology of hypertension. In this review, we have summarized the literature on electrolytes in preeclampsia by conducting an extensive systematic search of databases such as PubMed, Excerpta Medica database (EMBASE), and Medical Literature Analysis and Retrieval System Online (MEDLINE).

## Introduction and background

Preeclampsia, a complex multisystem disorder, affects approximately 3%-8% of pregnancies in Western countries and is a significant global contributor to maternal and perinatal morbidity and mortality. Preeclampsia and eclampsia are responsible for 10%-15% of maternal deaths. Both immunological and genetic theories of etiology are supported by epidemiological data. A history of preeclampsia in a mother increases the risk of subsequent pregnancies by two to five times. Ethnically, preeclampsia affects between 1% and 3% of multiparas and between 3% and 7% of healthy nulliparas. Nulliparity and a new partner have also been identified as risk factors [1]. Preeclampsia, a hypertensive disorder of pregnancy, has been reported with a higher prevalence of approximately 28% in some regions (like Sub-Saharan Africa, Latin America, and the Middle East), which is noted to be higher than the rest of the world [2]. Recent attention has been given to the examination of dietary factors in the development of preeclampsia. Research suggests that deficits in magnesium, calcium, and zinc during pregnancy are associated with intrauterine growth restriction (IUGR), preeclampsia, eclampsia, and preterm delivery [3]. The etiology of preeclampsia remains unknown, with ongoing investigation into its primary cause, including the role of pulmonary embolism [4]. The condition is expected to occur in two distinct stages: the first is characterized by the invasion of fetal trophoblastic decidua and local placental hypoxia, and the second is characterized by the release of placental factors into the maternal blood, along with the manifestation of pro-inflammatory, antiangiogenic, and angiogenic factors [5-7].

The International Society for the Study of Hypertension in Pregnancy (ISSHP) has established a definition for hypertensive disorders of pregnancy, which refers to the occurrence of elevated blood pressure (specifically, systolic blood pressure of ≥140 mmHg or diastolic blood pressure of ≥90 mmHg) that emerges after the 20^th^ week of gestation. This comprehensive categorization encompasses chronic hypertension (HTN), gestational HTN, and preeclampsia (either occurring independently or as a complication of chronic HTN) [8]. These conditions can have significant consequences for both maternal and fetal health, including an increased risk of long-term hypertension, cardiovascular death, serious adverse cardiovascular events, and stroke [9]. Theories of preeclampsia pathophysiology include both maternal and fetal variables. Although the exact etiology of preeclampsia is unknown. Evidence points to abnormal placental implantation and trophoblastic invasion. Additionally, research suggests that prenatal exposure to hypertensive disorders can lead to significant and lasting cardiovascular effects in offspring, such as early-onset HTN and increased susceptibility to ischemic heart disease and stroke [6,7,10]. Predicting preeclampsia is a significant challenge in clinical practice. The development of accurate prediction models to identify women at increased risk would enable targeted preventive measures, such as aspirin therapy, and intensified surveillance, thereby reducing adverse outcomes. Insufficient recognition of risk factors contributes to substandard healthcare, which is associated with maternal mortality [11]. Studies are exploring the efficacy of multiple-marker algorithms in predicting preeclampsia, similar to those used in first-trimester aneuploidy screening. Previous studies have shown variations in the concentrations of pregnancy-related plasma protein A (PAPP-A), disintegrin and metalloproteinase 12 (ADAM12), placental growth factor 8 (PlGF8), angiopoietin 1 and 2, inhibin A and activin A, and soluble endoglin during the first trimester of pregnancy [12,13].

## Review

Risk factors

Interpreting research on the correlation between racial background and preeclampsia risk requires caution due to the potential omission of crucial mediators and confounding factors. Comprehensive cohort research in the United Kingdom, involving over 168,000 women with singleton pregnancies, showed that Black women had a double likelihood of developing preeclampsia compared to White women. This risk was highest for early-onset preeclampsia, with a 3.5-times increase, followed by preterm preeclampsia, which showed a 2.5-times higher risk [14]. The use of the Fetal Medicine Foundation algorithm for first trimester screening has been found to significantly improve perinatal outcomes by 60% in Black populations, indicating that health disparities can be mitigated through individualized risk assessment and appropriate care pathways [15,16].

The relationship between preeclampsia and maternal age exhibits a J-shaped pattern, with increased risk for both adolescent mothers and women aged 35 years and older. Advanced maternal age is associated with an increased risk of multiple pregnancies, the use of assisted reproductive technologies, and pre-existing cardiometabolic abnormalities and medical problems, all of which can increase the risk of preeclampsia. Evidence suggests that the likelihood of developing preeclampsia increases with each successive year beyond the age of 32 [17,18]. Women under 20 years of age may have an increased risk of adverse pregnancy and childbirth outcomes due to physiological, immunological, and socioeconomic factors. Late-onset preeclampsia (occurring at or after 34 weeks of gestation) is primarily linked to a maternal age below 20 years [19]. Pre-existing health issues can increase the risk of hypertensive disorders such as preeclampsia. Risk factors identified before or early in pregnancy can allow for interventions to reduce the chance of developing preeclampsia later. Women with chronic HTN, defined as HTN diagnosed before the 20^th^ week of pregnancy, have a five-fold higher risk of developing preeclampsia [15,20]. Those with pre-gestational diabetes mellitus have a three-fold higher risk of preeclampsia compared to women without diabetes. These women may have pre-existing microvascular and macrovascular complications associated with diabetes, such as renal disease, which contributes to this increased risk. Diabetes can also contribute to endothelial dysfunction, inflammation, and oxidative stress, typical pathways linked to the pathophysiology of preeclampsia [21,22].

Women with chronic renal disease have a significantly higher incidence of preeclampsia. Conditions such as diabetes, glomerulonephritis, and polycystic kidney disease are associated with a higher risk, particularly for late-onset preeclampsia. The severity of renal disease and proteinuria levels are significant predictors of preeclampsia incidence, especially in the absence of concurrent chronic HTN [23]. Primiparity has been reported to triple the probability of experiencing preeclampsia [24]. Immunological maladaptation and maternal alloimmune responses, triggered by the rejection of paternal antigens in the fetal allograft, can contribute to preeclampsia [25]. Preeclampsia is most commonly diagnosed in early pregnancy, making primiparous mothers more susceptible. Conversely, multiparity acts as a protective factor, reducing the risk of preeclampsia [26]. This protective mechanism is nullified in future pregnancies involving exposure to new paternal antigens [27]. A meta-analysis of 94 studies revealed a 13.8% recurrence risk of preeclampsia, which is inversely related to the gestational age at delivery in the previous affected pregnancy [28,29]. Preeclampsia is also associated with the use of assisted reproductive technologies compared to naturally conceived pregnancies or those achieved through intrauterine insemination. Frozen-thawed embryo transfer cycles carry a higher risk of preeclampsia compared to fresh cycles, possibly due to the absence of a corpus luteum at conception, which may compromise maternal vascular health [30].

Pathophysiology

In a normal pregnancy, the villous cytotrophoblast (CTB) is known to invade the inner third of the myometrium. Additionally, the spiral arteries undergo a transformation, losing their endothelial lining and significantly reducing their muscle fiber content. This change converts them into low-resistance vessels, making them less responsive or even immune to vasoconstrictive drugs [31]. These anatomical alterations are accompanied by functional changes. The pathogenesis of preeclampsia is multifactorial, with abnormal placentation being a key underlying factor. During preeclampsia, the CTB cells inadequately colonize the spiral arteries. The two-stage model proposes that placental abnormalities due to decreased perfusion produce factors that contribute to maternal disease. Recent studies have shown that the invasion of the uterus by CTB involves a unique differentiation process where fetal cells acquire characteristics identical to the maternal endothelium, often replacing it. This differentiation process is disrupted in cases of preeclampsia [32]. The temporary blockage of maternal arteries by endovascular extra-villous trophoblast invasion restricts blood flow to the developing placenta, creating a low-oxygen environment that supports embryonic development. Around 10-12 weeks of gestation, the trophoblast endovascular plugs are shed, allowing increased maternal blood flow into the intervillous space, which enhances fetal oxygenation through the placenta [33,34].

**Figure 1:**
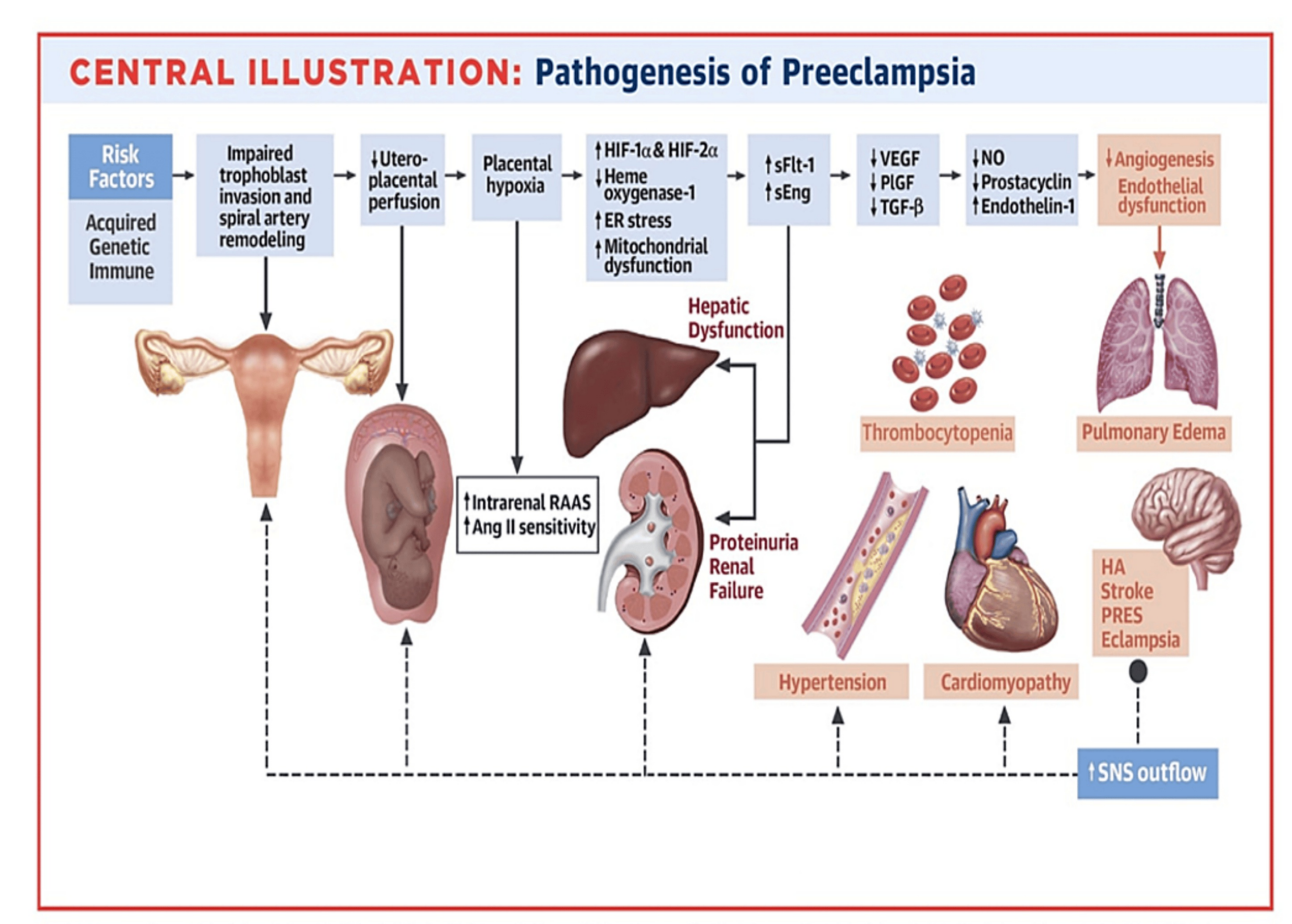
A two-stage model illustrating the cause and development of preeclampsia Acquired, genetic, and immune risk factors contribute to early placental dysfunction (Stage 1). Placental dysfunction results in the release of anti-angiogenic factors, leading to later multiorgan dysfunction (Stage 2). Solid arrows represent the progression of the disease. Dashed arrows represent the SNS effect on respective organs. Ang II: anqiotensin II; ER: endoplasmic reticulum; HA: headache; HIF: hypoxia-inducible transcription factor; NO: nitric oxide; PIGF: placental growth factor; PRES: posterior reversible encephalopathy syndrome; RAAS: renin-angiotensin-aldosterone system; Eng: endoglin; FIt: fms like tyrosine kinase; SNS: sympathetic nervous system; TGF: transforming growth factor; VEG: vascular endothelial growth factor Reprinted from the Journal of the American College of Cardiology, Volume 76, Ives CW, Sinkey R, Rajapreyar I, Tita ATN, Oparil S,  "Preeclampsia—Pathophysiology and Clinical Presentations: JACC State-of-the-Art Review", Pages 1690-1702, Copyright 2020, with permission from Elsevier [40].

The maternal cardiovascular system undergoes significant adaptation during pregnancy, marked by an increase in plasma volume and cardiac output, detectable as early as three to four weeks of gestation, largely influenced by placental factors [34]. The prevention of resultant increases in blood pressure is mediated by various mechanisms, including a decrease in systemic peripheral resistance, increased arterial compliance, promotion of peripheral vasodilation, reduced contractility, activation of the renal renin-angiotensin system, and increased production of vasodilatory substances by the endothelium. Information on the influence of autonomic regulation on circulatory changes during pregnancy is limited. The primary modifications observed in the cardiovascular system during pregnancy are mainly attributed to the endothelium and myogenic mechanisms. The endothelium, acting as an intermediary between blood and vascular smooth muscle, is highly responsive to both humoral stimuli and physical forces [35-37]. Histological changes in ,the placenta associated with preeclampsia and fetal growth restriction (FGR) suggest reduced perfusion, characterized by rapid villus branching, numerous and large syncytial knots, and small, sclerotic villi. These features are seen in term preeclampsia but are more typical of preterm preeclampsia and FGR [38-40]. A two-stage model illustrating the cause and development of preeclampsia is presented in Figure 1.

The clinical features observed in the mother, such as the HELLP (hemolysis, elevated liver enzymes, and low platelet count) syndrome, refractory neurological disorders, and eclampsia, can be attributed to endothelial dysfunction. Specifically, hepatic and cerebral endothelial dysfunction play a crucial role in the pathogenesis of HELLP syndrome and refractory neurological disorders with eclampsia, respectively [39]. Endotheliosis is exacerbated in conditions with deficient levels of vascular endothelial growth factor (VEGF), leading to reduced glomerular filtration and proteinuria. The malfunction of endothelial cells contributes to the development of microangiopathic hemolytic anemia.

A characteristic feature of preeclampsia is a failure in the mother's immunity, which impedes the normal recognition of the fetoplacental unit. Excessive proliferation of immune cells leads to the secretion of tumor necrosis factor-alpha, causing the death of extravillous cytotrophoblasts. The abnormal invasion of spiral arteries has been linked to the human leukocyte antigen (HLA) system, as women with preeclampsia have shown reduced expression of HLA-G and HLA-E. Natural killer (NK) cells typically release VEGF and placental growth factor, facilitating cellular interaction between these cells and the trophoblast in normal pregnancies [32,33]. In preeclamptic women, elevated levels of soluble FMS-like tyrosine kinase 1 (sFlt-1) act as an antagonist to placental and vascular endothelial growth factors [1]. Oxidative stress and angiogenesis pathways related to gene expression were found to be dysregulated in placental tissue. Additionally, altered genes associated with NK cell activity, cell movement, decidualization, and inflammation/immunoregulation were identified in the decidual tissue [40,41]. A meta-analysis of placental samples obtained before delivery revealed dysregulated genes linked to immunological response, development and pregnancy processes, and glucose and energy metabolism across all forms of preeclampsia [42].

Methodology

This systematic review adhered to the Preferred Reporting Items for Systematic Reviews and Meta-Analyses (PRISMA) guidelines. The literature search was conducted using Medical Subject Heading (MeSH) keywords, including 'preeclampsia', 'electrolyte', and 'derangement'. The databases searched included PubMed, the Institute for Scientific Information (ISI) Web of Science, the Excerpta Medica database (EMBASE), the Cochrane Library, Medical Literature Analysis and Retrieval System Online (MEDLINE), and Google Scholar, with no language restrictions. The search was limited temporally from the inception of each database until August 6, 2023. All search queries used a combination of subject-specific and free-text terms.

**Figure 2:**
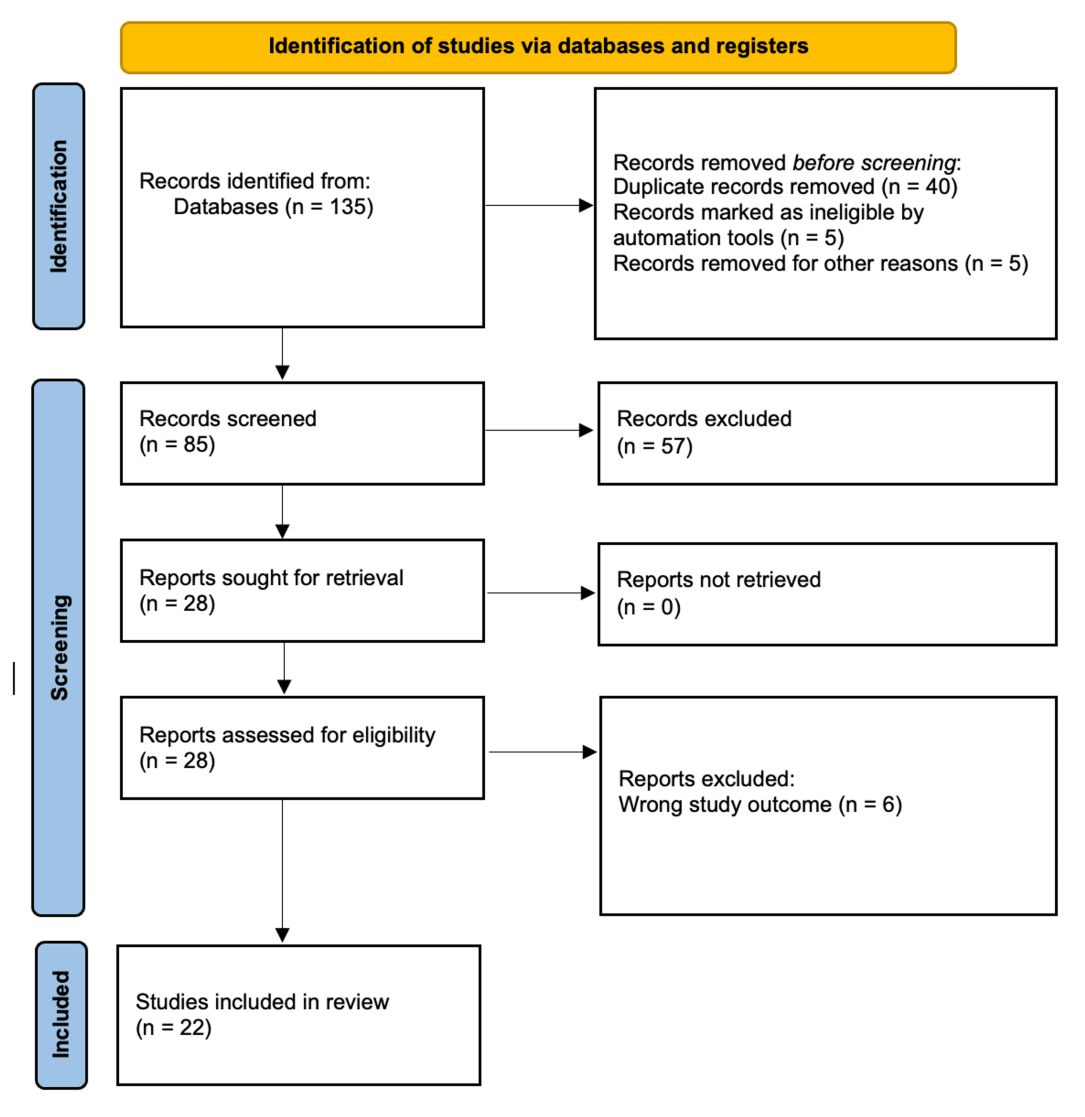
A PRISMA flowchart representing the data collection process PRISMA: Preferred Reporting Items for Systematic Reviews and Meta-Analyses

The PRISMA flowchart provides a visual representation of the data collection process, making it accessible and easy to follow and providing a clear overview of the complex procedure (Figure 2).

From the search, a total of 135 records were initially identified. After removing duplicates, 85 records remained. These records were screened, resulting in the exclusion of 57 records due to reasons such as unsuitable publication types (case reports, abstracts, different languages), incomplete data without original text, and overlap with other included studies. The remaining 28 full-text articles were assessed for eligibility. Out of these, six articles were excluded for specific reasons, leading to the final inclusion of 22 studies in the comprehensive review (Table 1).

**Table 1:** Studies included in the comprehensive review

Serial no.	Author	Year	Study parameters	Number of pregnancies
1	Uzan J et al. [1]	2011	Pathophysiology, diagnosis, and management of preeclampsia	Not specified (review article)
2	Jain S et al. [3]	2010	Role of calcium, magnesium, and zinc in preeclampsia	N = 50
3	Turpin CA et al. [4]	2015	Imbalance in angiogenic regulators and oxidative stress	N = 170
4	Davis EF et al. [10]	2012	Cardiovascular risk in children of preeclamptic pregnancies	N = 45249
5	Schneuer FJ et al. [11]	2014	Angiopoietin levels as biomarkers for adverse outcomes	N = 4985
6	Odibo AO, et al. [12]	2014	First-trimester biomarkers and adverse pregnancy outcomes	N = 193
7	Myatt L et al. [13]	2013	Changes in angiogenic biomarkers predicting preeclampsia	N = 626
8	Arechvo A et al. [14]	2022	Racial influence on preeclampsia	N = 168966
9	Liu BP et al. [16]	2022	First-trimester screening impact on perinatal mortality	N = 20651
10	Poon LC et al. [17]	2010	Multivariate analysis of maternal risk factors for hypertensive disorders	N = 8061
11	Yang Y and Wu N [22]	2022	Correlation between gestational diabetes and preeclampsia	N = 23316
12	Khalaf SA et al. [23]	2022	Chronic kidney disease and adverse pregnancy outcomes	Not specified (meta-analysis)
13	Collier AY et al. [25]	2021	Immune mechanisms and potential therapies for preeclampsia	Not specified (systematic review)
14	Robillard PY et al. [27]	1993	Placental and maternal preeclampsia	N = 1800
15	Robillard PY et al. [28]	2020	Validated the 34-week gestation as the definition of late-onset preeclampsia	N = 1700
16	Van Oostwaard MF et al. [29]	2015	Meta-analysis on the recurrence of hypertensive disorders in pregnancy	N = 99415
17	Opdahl S et al. [30]	2015	Cohort study on the risk of hypertensive disorders following assisted reproductive technology	N1 = 58006 (assisted reproductive technology pregnancies), N2 = 315273 normal pregnancies
18	Aplin JD et al. [34]	2020	Tracked placental development in health and disease	Not specified (meta-analysis)
19	Kumar S et al. [36]	2018	Implications of androgens in maternal vascular and placental function for preeclampsia pathogenesis	Not specified (review article)
20	Lumbers ER et al. [37]	2019	Dysregulated maternal renin-angiotensin system in preeclampsia	Not specified (review article)
21	Roberts DJ and Post MD [38]	2008	Placental involvement in preeclampsia	Not specified (systematic review)
22	Ives CW et al. [40]	2020	Pathophysiology and clinical presentations of preeclampsia	Not specified

Role of electrolytes in preeclampsia

Preeclampsia is characterized by increased salt retention, a natural phenomenon during pregnancy. The importance of electrolytes in the development of hypertension is well documented. Nutritional deficiencies in both macronutrients and micronutrients are a significant health concern in impoverished countries, particularly among women of reproductive age. Various dietary components, including protein, fats, magnesium (Mg2+), calcium (Ca2+), zinc, and copper, have been associated with an increased risk of preeclampsia [3]. Magnesium plays a crucial role in the physiological regulation of blood pressure due to its impact on vascular elasticity, contractility, and responsiveness. It is essential for the activation of several enzyme systems and has been implicated in both fetal and maternal morbidity during the prenatal and postnatal periods. Additionally, it is beneficial in the management of preeclampsia [3,43]. Magnesium acts as a Ca2+ channel antagonist, promoting the production of vasodilators such as prostacyclin and nitric oxide (NO) and altering vascular responses to vasoactive agonists. Hypertension pathophysiology is associated with Mg2+ deficiency, as evidenced by epidemiological and experimental studies showing an inverse relationship between blood pressure and serum Mg2+ levels [43,44]. Inadequate nutritional intake during pregnancy can be harmful to both the mother and the developing fetus. Fetal growth restriction is often a result of impaired placental function and is strongly correlated with preterm preeclampsia.

Late-onset preeclampsia, when complicated by FGR, is characterized by a decrease in placental weight and an increased presence of vascular and villous abnormalities compared to cases of late-onset preeclampsia without FGR [31,32]. The ISSHP recommends the use of ultrasonography to monitor fetal growth velocity, amniotic fluid volume, and umbilical artery Doppler every two weeks in pregnancies where FGR is suspected. However, the effectiveness of umbilical artery Doppler in the later stages of pregnancy may be limited. In low-resource settings, the ISSHP suggests assessing perinatal risk at a gestational age of 32 weeks or later by checking fetal heart rate, performing cardiotocography every six hours, and considering maternal characteristics along with the presence of proteinuria. At this gestational age, the primary risk determinant is the low gestational age itself [8]. Alterations in nutrient concentrations during pregnancy can affect pregnancy and delivery outcomes due to changes in maternal and conceptus metabolism. The levels of zinc and Mg2+ in the blood during both normal pregnancies and those affected by preeclampsia have been the subject of numerous clinical investigations. Throughout pregnancy, there is a consistent decrease in maternal serum levels of Mg2+, Ca2+, and zinc due to hemodilution, increased urine production, and enhanced transfer of these elements to the developing fetus. Calcium is essential for the normal function of vascular smooth muscles. Research has shown that blood Ca2+ and Mg2+ concentrations are associated with arterial relaxation in pregnant women [45]. Since low blood Ca2+ levels stimulate the production of renin and parathyroid hormones, which increase intracellular Ca2+ in vascular smooth muscle, blood Ca2+ levels appear to be critical in the pathophysiology of preeclampsia [46]. Furthermore, calcium supplementation has been shown to effectively reduce the risk of preeclampsia and its complications [47].

Electrolytes such as potassium (K+), sodium (Na+), Mg2+, and Ca2+ are important for the proper function of vascular smooth muscles, which can help prevent preeclampsia. Increased plasma concentrations of aldosterone and other mineralocorticoids may be responsible for the hypokalemic changes observed in normal pregnancy. Potassium deficiency in the body can result from inadequate K+ conservation by the kidneys and alimentary canal. In some cases, fecal loss of K+ may exceed urinary loss. In humans, 90% of the total body K+ is sequestered intracellularly, while Na+ is predominantly extracellular. This preferred Na+ and K+ distribution relies on the active transport of the Na+ pump. Lowered sodium-potassium cotransport activity may be an early sign of impaired Na+ and K+ transport across the vascular smooth muscle cell membrane [48]. Preeclampsia is associated with hypocalcemia, hypomagnesemia, hypokalemia, and hypernatremia, which may have a significant causal role. Adjuvant Ca2+, Mg2+, and K+ supplementation, along with Na+ restriction, may help prevent the progression of preeclampsia. Regular monitoring of serum Ca2+, Mg2+, Na+, and K+ levels may reduce the severity and sequelae of preeclampsia [43-47]. Preeclampsia may have a dual etiology of high Na+ and low K+ levels. Sodium and potassium may act as risk factors or predisposing variables, especially in susceptible individuals, rather than as primary causative factors. Excessive lipid peroxidation, or oxidative stress, has been linked to preeclampsia. There is an imbalance between the production of prooxidants and the activity of antioxidant enzymes. Altered antioxidant levels in human essential hypertension and preeclampsia suggest they may play a role in their pathogenesis. Zinc and Mg2+ are equally important for the proper functioning of enzymes that scavenge free radicals, such as superoxide dismutase. Deficiencies in these elements during pregnancy may impair cellular antioxidant capacity by reducing superoxide dismutase activity and increasing lipid peroxidation, thereby affecting blood pressure [43,45,48,49].

Discussion

Preeclampsia is associated with elevated rates of morbidity and mortality in both the fetal and maternal populations. Mothers with preeclampsia may have an increased risk of early cardiovascular diseases, such as ischemic heart disease, stroke, and persistent hypertension, later in life. Similarly, adults born with low birth weight and mothers with preeclampsia are at a higher risk of developing metabolic syndrome, stroke, and coronary heart disease [1,10]. It is crucial to focus on interventional studies during the asymptomatic postpartum period to reduce the incidence of potentially fatal cardiovascular diseases. Preeclampsia can also cause organ damage due to elevated blood pressure beyond 20 weeks of pregnancy, with delivery of the fetus and placenta being the only treatment, which can put the baby at risk [24]. 

Understanding the development and consequences of preeclampsia is essential for prevention. The clinical presentation, long-term prognosis, laboratory results, and response to prophylactic therapies vary among preeclampsia subtypes. Ca2+, Mg2+, and zinc may contribute to these variations. Since micronutrient levels in the body change dynamically during pregnancy, assessing these elements during gestation could aid in the early detection and treatment of preeclampsia. Pregnancy increases the demand for various minerals, including copper, zinc, vitamin B12, vitamin C, and others, to meet the nutritional needs of the developing fetus. These nutrients play a critical role in regulating development in both humans and animals due to their involvement in signal transduction pathways and the activity of multiple enzymes and gene transcription factors [3,43,45]. 

Changes in maternal and fetal metabolism during pregnancy can directly affect pregnancy outcomes, including pregnancy and delivery. Research by Pathak et al. and Wyn et al. has shown that Mg2+ supplementation during pregnancy can reduce the risk of preterm delivery and intrauterine growth retardation [43,50]. Arumanayagam M et al. reported that a decrease in sodium-potassium cotransport activity during pregnancy-induced hypertension may be an early sign of impaired Na+ and K+ transport across vascular smooth muscle cell membranes, which is essential for blood pressure regulation [51]. 

Sukonpan et al.'s research revealed increased glutathione peroxidase and decreased antioxidant levels, such as catalase and superoxide dismutase, in preeclampsia patients, along with elevated plasma levels of nitric oxide and lipid peroxides [46]. The Optimizing Titration and Monitoring of Maternal Blood Pressure (OPTIMUM) randomized controlled trial evaluated adherence and self-reported and clinical blood pressure readings, showing a small difference in readings with good adherence among participants [52]. However, some poorly understood clinical symptoms, such as liver dysfunction, hematologic abnormalities, superimposed eclampsia, and cardiac remodeling and dysfunction, are characteristics of preeclampsia [2,12,14,15]. Despite the well-studied clinical significance of preeclampsia due to its substantial short- and long-term effects on maternal and neonatal morbidity and mortality, timely diagnosis and management are crucial for a better prognostic outcome.

## Conclusions

The etiology and pathogenesis of this complex disorder remain unclear, as the underlying mechanisms and origins of the syndrome are yet to be fully understood. While current research on placental oxidative stress and the maternal immune response yields positive results, the most promising field of study is likely the imbalance of maternal angiogenic factors and their implications for vascular function. Significant progress has been made in reducing maternal and fetal morbidity and mortality, largely attributed to improved clinical knowledge and care. Further understanding of placental development, its interactions with the uterine lining, and its pathophysiology during pregnancy is necessary to complement current achievements and enhance prediction and prevention.
